# The ecological factors contributing to the chikungunya outbreak in Ruili, a border city in Yunnan Province, China

**DOI:** 10.1186/s12879-025-12056-1

**Published:** 2025-11-24

**Authors:** Yu-Feng Yang, Xin-Lou Li, Ting-Song Hu, Xiao-Xiong Yin, Qiang Xu, Yao Tian, Yong-Hua Liu, Ping Li, Si-Yu Chen, Zhao-Lan Yang, Hai-Feng Pan, Chen-Long Lv, Guo-Lin Wang, Fu-Qiang Zhang, Wei Liu, Li-Qun Fang

**Affiliations:** 1 Department of Epidemiology and Health Statistics, School of Public Health, Anhui Medical University, Hefei, 230032 P. R. China; 2 State Key Laboratory of Pathogen and Biosecurity, Academy of Military Medical Science, Beijing, 100071 P. R. China; 3 Department of Medical Research, Key Laboratory of Environmental Sense Organ Stress and Health of the Ministry of Ecology and Environmental of the People’s Republic of China, The Ninth Medical Center, Chinese PLA General Hospital, Beijing, P. R. China; 4 Center for Disease Control and Prevention of Southern Theater Command, Guangzhou, P. R. China; 5 Ruili Center for Disease Control and Prevention, Ruili, P. R. China

**Keywords:** Chikungunya fever, Chikungunya virus, Ecological analysis, Epidemiology

## Abstract

**Background:**

Chikungunya virus (CHIKV), an arthropod-borne pathogen, has caused recurrent epidemics in tropical and subtropical regions, as well as sporadic outbreaks in certain temperate areas, posing significant public health challenges. The globalization-driven increase in international tourism and trade has facilitated the spread of the virus, leading to several outbreak events in China triggered by imported cases, including the 2019 outbreak in Ruili City, Yunnan Province.

**Methods:**

This study conducts a quantitative analysis of the spatiotemporal patterns of outbreak in Ruili City and investigates the impact of ecological factors on the occurrence of local chikungunya cases and the extent of its autochthonous transmission, using panel logistic regression and Poisson regression models, respectively.

**Results:**

This outbreak began on September 20, 2019, and lasted for approximately twelve weeks, during which a total of 139 local infections and 31 imported infections were reported. The peak incidence occurred in the third week of the outbreak, after which a gradual decline was observed. Most cases were concentrated in five specific townships located in close proximity to the Jiegao Port. Modeling analysis revealed that the presence of imported cases, population density, and the percentage coverage of grassland were the most significant factors influencing autochthonous transmission of CHIKV, significantly influencing both the occurrence of local chikungunya cases and the extent of autochthonous transmission. Furthermore, the occurrence of local cases was positively associated with the presence of local cases reported in the previous week and precipitation levels in the week before last.

**Conclusions:**

Our findings suggest that autochthonous transmission of chikungunya fever following case importation is significantly associated with imported case, high population density, and areas with great grassland coverage. A comprehensive understanding of the factors facilitating autochthonous transmission can enhance future surveillance efforts, environmental management strategies, and disease control measures for chikungunya fever.

**Supplementary Information:**

The online version contains supplementary material available at 10.1186/s12879-025-12056-1.

## Background

Chikungunya fever is an acute, mosquito-borne viral disease caused by the chikungunya virus (CHIKV). The incubation period typically ranges from 3 to 7 days. The acute phase is characterized by common clinical manifestations including fever, rash, arthralgia, myalgia, headache, and nausea [1]. Given the substantial overlap of these symptoms with those observed in dengue and Zika virus infections, differential diagnosis can be challenging and misdiagnosis may occur [2]. Although neurological complications are relatively uncommon, they tend to be severe and predominantly affect pediatric and geriatric populations [3]. Around 40% to 80% of patients transition into a post-acute chronic phase, with about 25% continuing to experience persistent joint pain after a year. This prolonged morbidity often results in diminished mobility and a significant decline in quality of life [4]. In pregnant women, there is a risk of vertical transmission of CHIKV from month to child, with an estimated transmission rate of approximately 15%; this increases to nearly 50% when infection occurs during the perinatal period [5]. The overall risk of neonatal death associated with maternal infection is 0.6%, rising to 2.8% in cases of neonatal infections [4].

In 1952, CHIKV was first identified on the Makonde Plateau in East Africa and subsequently reported in various countries across Africa and Asia [3]. To date, local CHIKV cases have been reported in 119 countries and territories worldwide [6]. Significant outbreaks have recently occurred across Africa, Asia, the Indian Ocean region, and more recently in the Caribbean and the Americas [7]. Globalization-driven population mobility has significantly influenced the global distribution of infectious diseases and posed challenges to public health systems. Between 2014 and 2018, China reported 3,128 imported vector-borne infections among inbound travelers, including 13 cases of chikungunya fever [8]. Predictive models suggest a considerable likelihood of *Aedes aegypti* presence in southern China, particularly in Yunnan, Guangdong, Hainan, and Guangxi provinces. In contrast, the distribution of *Aedes albopictus* is projected to extend across a significantly broader geographic range within the country [9]. In China, CHIKV was first isolated from bat (*Rousettus leschenaulti*) brain tissue in 1986 in Yunnan Province, followed by isolation from a febrile patient in Yunnan in 1987 [10], indicating the potential existence of natural foci in this region. An outbreak of chikungunya fever occurred in September 2010 in Dongguan and Yangjiang cities, Guangdong Province [11], resulting in 253 reported cases. Another outbreak in 2017 resulted in four confirmed cases in Quzhou City, Zhejiang Province [12]. The risk of CHIKV transmission in China remains substantial due to frequent importation of cases via international travelers, the widespread presence of competent mosquito vectors, and the general population’s susceptibility to infection.

CHIKV is predominantly transmitted by *Aedes aegypti* and *Aedes albopictus* mosquitoes, which are primarily distributed in tropical and subtropical regions [4]. The expansion of international travel and global trade, coupled with the intensifying impacts of climate change, has facilitated the spread of *Aedes* mosquitoes into an increasing number of temperate regions. Furthermore, increasing levels of human mobility driven by globalization have increased the likelihood of individuals carrying the virus across borders, thereby amplifying the potential for viral introduction and local transmission [4, 13]. In China, the specific transmission patterns and ecological determinants underlying these cross-border and local outbreaks remain unclear. An in-depth and comprehensive analysis of the spatiotemporal patterns and ecological factors would enhance understanding of autochthonous transmission following disease importation and significantly contribute to the development of more effective surveillance and control strategies.

This study sheds light on the spatiotemporal dynamics of both imported and autochthonous cases during the CHIKV outbreak in Ruili City in 2019 and quantifies the effect of ecological factors on disease transmission. These findings may inform future surveillance, prevention and control strategies for mitigating autochthonous transmission following its importation.

## Methods

### Study area

**Fig. 1:**
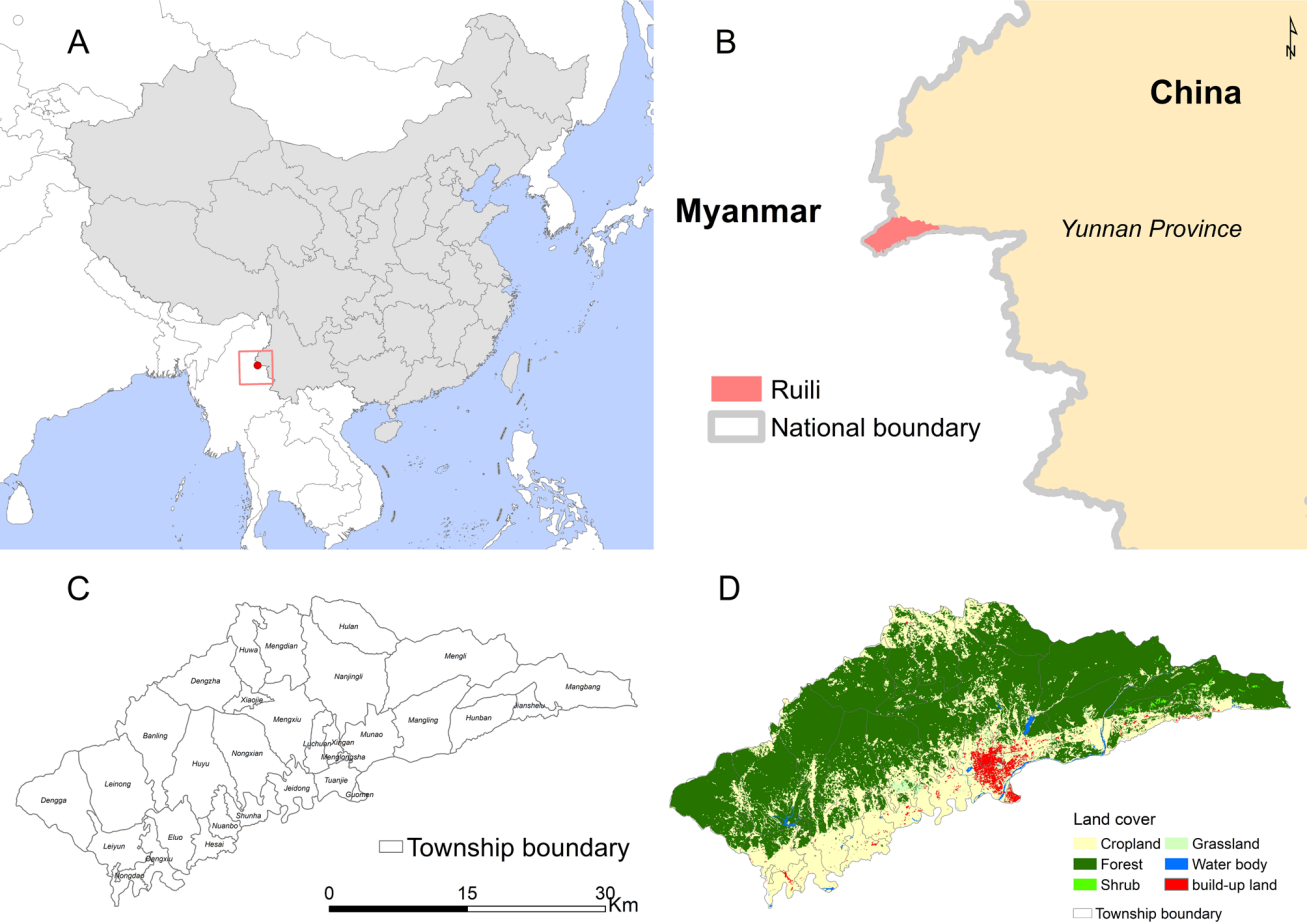
Maps of the study area. (**A**) The location of Ruili City, Yunnan Province in China. (**B**) The specific location of Ruili City in Yunnan Province. (**C**) The townships of Ruili City. (**D**) The land cover of Ruili City

Ruili City, located in Yunnan Province, lies on the southwestern border of China (Fig. 1A) and shares a contiguous boundary with Myanmar (Fig. 1B). Covering an area of approximately 1,000 km^2^, the city had a population of over 234,000 as of 2023. Administratively, Ruili City comprises 31 townships (Fig. 1C). The landscape is predominantly characterized by forested and agricultural land, which constitute the primary land cover types across the study area (Fig. 1D).

### Surveillance data

Data on the chikungunya outbreak were retrospectively collected from the Ruili Center for Disease Control and Prevention (Ruili CDC). The case definitions were established in accordance with the National Health Commission’s Guidelines for Chikungunya Fever (No. WS/T 590–2018) [14]. A probable case was defined as an individual presenting with fever, arthralgia, and rash, with a history of travel to a chikungunya-endemic area or exposure to CHIKV within 12 days prior to symptom onset, and for whom dengue fever or other febrile diseases associated with rash had been excluded. A clinically diagnosed case was defined as an individual fulfilling the criteria for a probable case and demonstrating the presence of virus-specific IgM antibodies in a single acute-phase serum specimen. A confirmed case of CHIKV infection was defined as an individual meeting the criteria for either a probable or clinically diagnosed case, along with laboratory-confirmed CHIKV infection based on one or more of the following diagnostic criteria: a four-fold or greater increase in specific antibody titers (IgG or neutralizing antibodies) in convalescent-phase serum compared to acute-phase serum; seroconversion evidenced by a negative test in acute-phase serum and a positive result in convalescent-phase serum; isolation of CHIKV from an acute-phase serum sample; or detection of CHIKV RNA in an acute-phase serum sample. An imported confirmed case was defined as a conformed case with a travel history to countries or regions experiencing ongoing chikungunya fever outbreaks within 12 days before symptom onset. In the absence of such travel history, cases were classified as locally confirmed. Given the dengue outbreak in Yunnan Province in 2019 [15], all cases included in this study were confirmed to be free of dengue infection through NS1 antigen testing at the time of medical consultation. A total of 118 cases were identified via the detection of viral RNA in acute-phase sera using RT-PCR, and an additional four cases were identified based on the presence of virus-specific IgM antibodies in a single serum sample collected during either the acute or convalescent phase.

The Breteau Index (BI), defined as the number of containers positive for *Aedes* mosquito larvae per 100 houses surveyed, is an effective indicator for monitoring the population dynamics of *Aedes* species and assessing the effectiveness of mosquito control interventions [16]. A higher BI suggests a greater number of potential breeding sites; however, actual adult mosquito abundance is also influenced by various factors, including larval mortality, pupal survival, adult longevity, and environmental conditions. Since 2014, the National Health Commission of the People’s Republic of China has implemented guidelines and a national surveillance system for *Aedes* mosquito monitoring, covering all provinces, autonomous regions, and municipalities in the mainland of China. Due to historical outbreaks of dengue fever, Ruili has been designated as a site for routine *Aedes* mosquito surveillance. Routine surveillance activities were conducted at multiple predetermined locations across Ruili City, with sampling sites selected based on a high risk of *Aedes* mosquito abundance or dengue transmission. In accordance with the Guidelines for the Control and Prevention of Dengue and Chikungunya fever, emergency *Aedes* mosquito surveillance was also implemented. During the CHIKV outbreak, personnel from the Ruili Center for Disease Control and Prevention conducted emergency surveillance in the core epidemic-affected areas between September 20 and December 29, 2019. This study utilized *Aedes* mosquito surveillance data collected from both routine and emergency surveillance activities. To effectively mitigate the chikungunya outbreak, a comprehensive and multifaceted strategy was performed, focusing on both vector control and the prevention of case importation. The Ruili CDC implemented targeted interventions to reduce mosquito populations in areas where chikungunya cases had been confirmed, as well as in surrounding zones associated with human activity.

### Other data

A township-level digital map of Ruili City was obtained from the Chinese Data Sharing Infrastructure of Earth System Science (http://www.geodata.cn/). Daily meteorological data, including temperature, relative humidity, and precipitation, were obtained from the Meteorological Bureau of China. Normalized Difference Vegetation Index (NDVI) data at a spatial resolution of 250 m × 250 m were collected from EarthData (https://search.earthdata.nasa.gov/search). Elevation data at a spatial resolution of 30 m × 30 m were collected from the SRTM Data repository (http://srtm.csi.cgiar.org/srtmdata/). Population density data at a spatial resolution of 100 m × 100 m were retrieved from WorldPop (https://www.worldpop.org/). Land-cover data at a spatial resolution of 30 m × 30 m were obtained from the National Cryosphere Desert Data Center (http://www.ncdc.ac.cn). Spatial analyst tools in ArcGIS 10.7 software (ESRI Inc., Redlands, CA, USA), including zonal calculates, were used to calculate the average values of NDVI, elevation, and population density, as well as the percentage coverage of grassland, built-up land, shrubs, cropland, forest, and water body at the township level. All cases were geo-referenced and linked to the digital map of Ruili City based on the residential locations at the time of symptom onset, utilizing GIS technologies.

### Statistical analysis

To characterize the epidemic dynamics of imported and locally transmission cases during the 2019 chikungunya outbreak in Ruili City, demographic characteristics of confirmed cases were summarized. Epidemic curves were created by plotting the weekly number of confirmed and probable cases based on the date of symptom onset, stratified by transmission origin (imported vs. local), to display the temporal dynamics of the outbreak. Weekly meteorological variables, including temperature, relative humidity, and precipitation, were also presented to contextualize environmental conditions during the outbreak period. To assess the epidemic situation, the weekly average BI of the whole Ruili city from emergency *Aedes* mosquito surveillance was displayed. We investigated the association between weekly average BI and the weekly meteorological variables with a lagged duration of 0 to 2 weeks in Ruili City by Spearman’s rank correlation analysis. The weekly average BI for the entire city was calculated based on routine *Aedes* mosquito surveillance data collected from 2018 to 2019 (see Table S1 and Figure S2). Spatial distributions of confirmed and probable cases (both local and imported) were mapped, and attack rates for each township were calculated and integrated into the spatial visualization.

### Panel logistics regression

To identify ecological factors associated with the occurrence of local cases, a panel logistic regression model was developed at the township level with a weekly temporal resolution. All ecological variables incorporated in the model, including the occurrence of local cases in the previous week [17, 18], the occurrence of imported cases [19], population density [20], NDVI, and elevation [21], were measured on a weekly basis during the outbreak period. Weeks with at least one local case were defined as “case” weeks, while weeks with no reported local cases were defined as “control” weeks. Additionally, land cover and meteorological variables were also incorporated into the analysis [20]. Meteorological factors generally exert a lagged effect on mosquito-borne diseases [22], influencing both mosquito population dynamics and human behavioral patterns. Based on previous studies [22], we retained the meteorological variables lagged zero to three-week separately into the model accounting for the lagged effect on mosquito-borne diseases [22]. Models were optimized by comparing − 2 log likelihood, and the final model was determined using the Akaike Information Criterion (AIC). A Hausman test was first conducted to assess the appropriateness of the fixed-effects (FE) versus random-effects (RE) model specification. The resulting chi-square statistic of 3.39 yield a p-value of 0.336, indicating no significant systematic differences between the two sets of estimates. Given the presence of repeated observations within townships over time, a random-effects panel logistic regression model was conducted to examine the association between the occurrence of local cases and ecological factors. Univariate analysis was initially performed to examine the independent effect of each variable. Odds ratios (ORs), their corresponding 95% confidence intervals (CIs), and p-values were estimated using maximum likelihood estimation. Subsequently, multivariate analysis was conducted by incorporating variables with a p-value < 0.20 from the univariate analysis as covariates.

### Poisson regression

The attack rate was calculated at the township level, and Poisson regression model was developed to explore the association between the attack rate and potential ecological factors. The dependent variable was defined as the attack rate per township, serving as an indicator of the scale of local transmission. Independent variables included the presence of imported cases during the study period (categorical), population density, NDVI, elevation, and the percentage coverage of various land-use types, such as grassland, built-up areas, shrub, cropland, forests, and water body. Initially, univariate analyses were performed to identify variables with a p-value less than 0.20. Subsequently, a variance inflation factor (VIF) test was carried out on the pre-selected variables. Only variables exhibiting a VIF value below 10 were incorporated into the multivariate analysis for further evaluation.

All the statistical analyses in this study were performed using the STATA software (Stata Corp LP, College Station, TX). A p-value less than 0.05 was considered statistically significant.

## Results

### Case characteristics

**Fig. 2:**
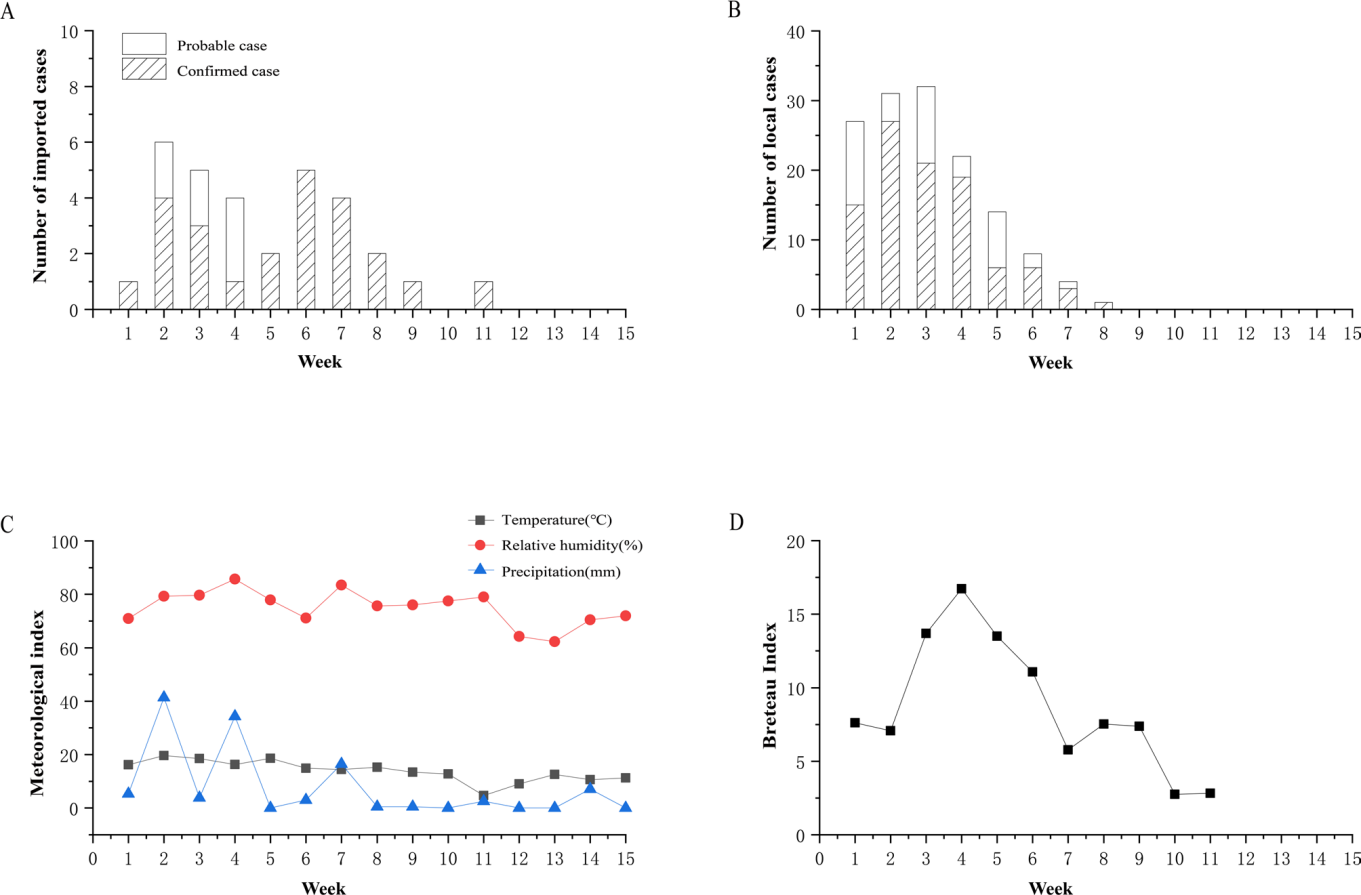
Temporal distribution of cases, meteorological variables and Breteau Index during the Chikungunya fever outbreak in Ruili City (Since September 20, 2019). (**A**) Imported confirmed and probable cases. (**B**) Local confirmed and probable cases. (**C**) Weekly average temperature, relative humidity and cumulative precipitation. (**D**) Weekly Breteau Index (BI) from the emergency monitoring

A total of 170 infection cases were identified between September 20, 2019, and December 4, 2019. Among these, 31 infections were classified as imported infections, including 24 confirmed and seven probable cases, while 139 infections were categorized as local infections, comprising 98 confirmed and 41 probable cases (Figure S1). The onset of symptoms in the index case on September 20 marked the initiation of the outbreak. Throughout the outbreak period, imported infections exhibited a continuous occurrence pattern with dual peaks (Fig. 2A), while local infections peaked during the third week (Fig. 2B). Following this peak, the number of local cases gradually declined, indicating a diminishing transmission intensity. During the outbreak, the weekly average temperature ranged predominantly between 10℃ and 20℃. In the initial weeks, rainfall of varying intensity occurred accompanied by high relative humidity, which exceeded 70% in most instances. The BI remained above the safety threshold of five until the tenth week (Fig. 2C, D) [23]. The results of Spearman’s rank correlation analysis revealed that temperature and precipitation with a two-week lag exhibited a stronger correlation with BI (Table S1).

**Fig. 3:**
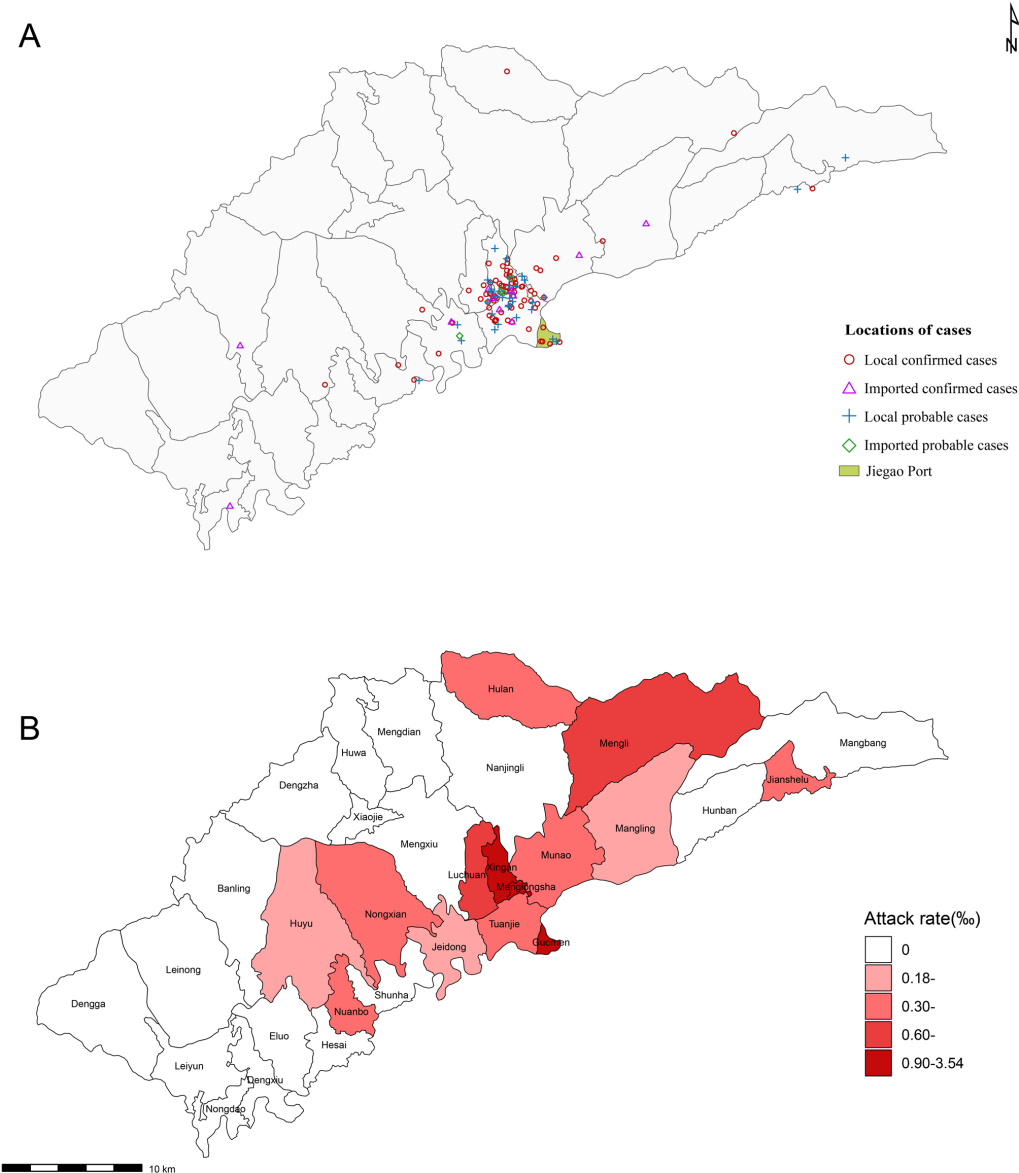
Spatial distribution of cases and attack rate at the township level during the outbreak. (**A**) Local and imported cases. (**B**) Attack rate

Among all patients, the proportion of females exceeded that of males, with approximately 80% of the total patient population aged between 18 and 59 years (Table 1). The highest concentration of cases was observed in townships located in close proximity to the Jiegao Port (Fig. 3A). Of the local cases, 81 were reported across five townships, including Tuanjie, Munao, Xingan, Menglongsha, and Luchuan, accounting collectively for 82.7% of all local cases. Similarly, 20 imported cases were distributed across these same five townships, representing 83.3% of the total imported cases. These townships are characterized by their geographical closeness to the port and high population density, both of which increase the likelihood of disease transmission (Table 1; Fig. 3A). The attack rate, defined as the number of local cases per 1,000 individuals, ranged from 0.18 to 3.54 cases per 1,000 persons (Fig. 3B).

**Table 1:** Demographic characteristics of confirmed cases in the 2019 Chikungunya outbreak in Ruili City, China

Variables	Local case				Imported cases	
	No. of cases	Percentage (%)			No. of cases	Percentage (%)
Sex						
Female	58	59.18			11	45.83
Male	40	40.82			13	54.17
Age (years old)						
0‒5	4	4.12			2	8.33
6‒17	6	6.19			3	12.50
18‒59	77	79.38			15	62.50
>60	10	10.31			4	16.67
Residence (township)						
Tuanjie	19	19.39			3	12.50
Munao	19	19.39			1	4.17
Xingan	17	17.35			12	50.00
Menglongsha	14	14.29			1	4.17
Luchuan	12	12.24			3	12.50
Others	17	17.35			4	16.67

### The association between the occurrence of local cases and ecological factors

After retained environmental factors and the meteorological variables lagged zero to three-week separately into the model, the final model was determined by the Akaike Information Criterion and presented in Table 2. In the univariate analysis of the panel logistic regression model, the occurrence of local cases in the previous week, the occurrence of imported cases, population density, NDVI, elevation, percentage coverages of grassland, built-up land, forest, and water body, as well as three meteorological variables (weekly average temperature with two lagged weeks, relative humidity, and cumulative precipitation with two lagged weeks) were significantly associated with local chikungunya fever occurrence (Table 2). Following stepwise variable selection in the multivariate logistic regression model, five variables remained statistically significant effect on local chikungunya fever occurrence. Specifically, local chikungunya fever occurrence was significantly associated with the occurrence of local cases in the previous week (OR = 19.728, 95% CI: 7.524‒51.724), the occurrence of imported cases (OR = 4.447, 95% CI: 1.068‒18.515), population density (OR = 1.066, 95% CI: 1.021‒1.114), percentage coverage of grassland (OR = 1.146, 95% CI: 1.033‒1.271), and weekly cumulative precipitation with two lagged weeks (OR = 1.171, 95% CI: 1.104‒1.242).

**Table 2:** The ecological analysis for the occurrence of local cases at Township level in Ruili City by the panel logistic model

Variable (unit)	Univariate analysis			Multivariate analysis		
	Crude OR	95% CI	*P* value	Adjusted OR	95% CI	*P* value
Occurrence of local cases in the previous week (categorical)	24.290	8.194, 72.009	< 0.001	19.728	7.524, 51.724	< 0.001
Occurrence of imported cases (categorical)	6.734	1.893, 23.956	0.003	4.447	1.068, 18.515	0.029
Population density (100 per km^2^)	1.138	1.079, 1.201	< 0.001	1.066	1.021, 1.114	0.004
NDVI	0.007	0.000, 0.134	0.001			
Elevation (m)	0.996	0.992, 1.000	0.043			
Land cover (%)						
Percentage coverage of grassland	1.356	1.184, 1.553	< 0.001	1.146	1.033, 1.271	0.010
Percentage coverage of built-up land	1.053	1.027, 1.080	< 0.001			
Percentage coverage of shrub	0.336	0.037, 3.046	0.332			
Percentage coverage of cropland	0.996	0.967, 1.026	0.807			
Percentage coverage of forest	0.973	0.954, 0.991	0.004			
Percentage coverage of water body	1.778	1.113, 2.841	0.016			
Meteorological factors						
Temperature (5 ℃)^*^	2.322	1.391, 3.876	0.001			
Relative humidity (10%)	1.805	1.004, 3.244	0.049			
Precipitation (5 mm)^**^	1.131	1.072, 1.945	< 0.001	1.171	1.104, 1.242	< 0.001

Abbreviations: CI, confidence interval; OR, odds ratio; NDVI, Normalized Difference Vegetation Index

^*^Temperature with two lagged weeks

^**^Precipitation with two lagged weeks

### The association between the extent of autochthonous transmission and ecological factors

In the univariate Poisson regression analysis, significant predictors (*p* < 0.05) included the occurrence of imported cases, population density, NDVI, elevation, and the percentage coverages of grassland, built-up land, shrub, forest, and water body. Following multivariate regression analysis of these variables that were significant in the univariate model, the results reveal that the extent of autochthonous transmission was significantly associated with three ecological variables: the occurrence of imported cases (IRR = 4.271, 95% CI: 2.167‒8.419), population density (IRR = 1.712, 95% CI: 1.334‒2.197), percentage coverage of grassland (IRR = 1.174, 95% CI: 1.116‒1.234) (Table 3). To address potential bias due to missing BI surveillance data, a sensitive analysis was conducted using data from townships where BI investigation was available. The results of sensitive analysis showed consistency with the primary Poisson regression model that included all townships (Table S2). The analysis revealed a similar result to the Poisson regression when all townships were included. While the univariate sensitivity analysis exhibited a significant association between BI and the extent of local transmission, this association did not remain statistically significant in the multivariate model.

**Table 3:** The ecological efficiency for the scale of local Chikungunya transmission at township-level in Ruili by Poisson regression model

Variable (unit)	Attack rate per 10,000(95% CI)	Univariate analysis			Multivariate analysis		
		Crude IRR	95% CI	*P* value	Adjusted IRR	95% CI	*P* value
The occurrence of imported cases (categorical)		14.667	8.329, 25.826	< 0.001	4.271	2.167, 8.419	< 0.001
No	1.989 (1.978, 1.999)						
Yes	6.716 (6.702, 6.731)						
Population density (1000 per km^2^) (continuous)		2.773	2.380, 3.230	< 0.001	1.712	1.334, 2.197	< 0.001
Population density (1000 per km^2^) (categorical)							
<0.1	1.145 (1.132, 1.158)						
0.1‒0.2	1.661 (1.642, 1.680)						
>0.2	6.084 (6.072, 6.097)						
NDVI		0.001	0.000, 0.003	< 0.001			
Elevation (m)		0.942	0.925, 0.960	< 0.001			
Land cover (%)							
Percentage coverage of grassland (1%) (continuous)		1.275	1.224, 1.328	< 0.001	1.174	1.116, 1.234	< 0.001
Percentage coverage of grassland (1%) (categorical)							
<1	1.068(1.060, 1.077)						
1‒10	5.463 (5.450, 5.476)						
>10	16.159 (16.095, 16.224)						
Percentage coverage of built-up land (1%) (continuous)		1.037	1.030, 1.043	< 0.001			
Percentage coverage of shrub (1%) (continuous)		0.131	0.019, 0.927	0.042			
Percentage coverage of cropland (1%) (continuous)		1.000	0.993, 1.007	0.985			
Percentage coverage of forest (1%) (continuous)		0.970	0.962, 0.978	< 0.001			
Percentage coverage of water body (1%) (continuous)		1.714	1.552, 1.894	< 0.001			

Abbreviations: CI, confidence interval; IRR: Incidence Rate Ratio; NDVI, Normalized Difference Vegetation Index

## Discussion

Chikungunya fever is transmitted between humans via the bites of infected *Aedes* mosquitoes, primarily *Aedes aegypti* and *Aedes albopictus* [24]. In China, CHIKV was first isolated from bat brain tissue in 1986. Subsequent outbreaks were documented in Guangdong Province in 2010 and Zhejiang Province in 2017. In the context of global climate warming, ecological shifts, and increased international travel and trade, CHIKV has expanded significantly across tropical and subtropical regions, leading to recurrent outbreaks of chikungunya fever. To date, 119 countries and territories have reported local cases of chikungunya fever, with more than 10 million documented infections and an estimated 1.3 billion people at risk of infection globally [6]. Climate modeling projections indicate that additional geographic regions may become suitable for CHIKV transmission in the future [25]. Beyond the primary symptoms of fever, rash, and arthralgia, acute CHIKV infection can present with a range of additional clinical complications, including neurological, cardiovascular, and respiratory disorders. Notably, arthralgia, which affects approximately 87% of acute cases, can progress to chronic arthritis in 40‒80% of patients [4], persisting for years and significantly impairing quality of life. According to a comprehensive review [26], CHIKV is associated with an average annual global burden exceeding 106,000 Disability-Adjusted Life Years (DALYs).

We provide quantitative analyses of local CHIKV cross-border transmission mechanisms in Ruili 2019, and interplay the case importation, vectors, environmental factors, and population. Our findings indicated that the occurrence of local chikungunya fever cases is associated with multiple factors, including the occurrence of imported cases, the presence of local cases in the previous week, population density, percentage coverage of grassland, and precipitation levels in the week before last. Infected individuals exhibit high viral loads, making them a primary source of transmission; thus, infected travelers can introduce the CHIKV across borders and seed transmission in previously unaffected areas [27]. Due to its geographical proximity to Myanmar, Ruili City faces a heightened risk of CHIKV transmission, primarily driven by frequent cross-border human movement, especially following a significant outbreak in Myanmar in 2019 [28, 29]. Most reported cases concentrated in the vicinity of the Jiegao Port. Given Yunnan’s status as a border region with a high potential for case importation, it is crucial to enhance the surveillance of inbound travelers and reinforce early warning systems. Furthermore, systematic exchange of epidemiological data with neighboring countries, such as Myanmar and Thailand, is essential to ensure timely and effective preventive measures. The predominate vectors of CHIKV, *Aedes albopictus* and *Aedes aegypti*, are known to thrive in environments with small water containers, and their proliferation is significantly exacerbated in densely populated areas characterized by high grassland coverage and substantial rainfall [30]. *Aedes* mosquitoes are free to proliferate without much control interventions, especially in abandoned land cover types such as neglected grasslands [31]. Our models demonstrated the significant role of grasslands in CHIKV transmission, which aligns with findings from previous studies [31]. Grasslands may serve as suitable reservoir for *Aedes* mosquitoes. Grasslands have been associated with favorable conditions for *Aedes* mosquito breeding [32], other findings that had also been reported in studies conducted in Italy and Florida [33, 34]. Higher population densities may increase the frequency of human-mosquito contact, especially during outdoor activities that coincide with the daily biting periods of *Aedes* Species [34]. Therefore, in addition to mitigating risks associated with imported cases, it is essential to implement comprehensive measures for mosquito control and eradication.

The extent of the autochthonous transmission of CHIKV is associated with the occurrence of imported cases, population density, and percentage coverage of grassland. Although Poisson regression analysis did not reveal a statistically significant association between the Breteau Index and the extent of autochthonous transmission, this may be attributed to limitations in data availability. The epidemic peaked during the third week and subsequently declined, while the Breteau Index peaked in the fourth week before sharply decreasing. The decline in the Breteau Index closely corresponds to the intensity of vector control interventions. As mosquito control measures were sustained and environmental conditions, particularly rainfall, remained unfavorable for mosquito breeding, the Breteau Index decreased significantly by the ninth week and remained below the safety threshold of 5 in the tenth and eleventh weeks [23]. Notably, although imported cases were reported in the ninth and eleventh weeks, no local transmission was observed during these periods.

Our results indicate that the occurrence of imported cases and population density are significant determinants of both local case incidence and the extent of autochthonous transmission of CHIKV. These findings underscore the necessity of enhancing surveillance of inbound travelers and implementing targeted prevention and control measures in areas with high population density. Furthermore, the presence of local cases in the preceding week is positively associated with the incidence of subsequent local cases, emphasizing the critical role of timely disease surveillance and early intervention. Prompt identification and effective management of local cases are essential to curbing further transmission. Moreover, environmental factors such as grassland coverage and precipitation are significantly associated with case occurrence, likely due to their impact on creating favorable breeding habitats for mosquito vectors. This reinforces the need for robust vector control strategies as part of integrated public health efforts. Foshan and Jiangmen City in Guangdong Province, southern China, with subtropical climate, has recently reported an ongoing local outbreak of chikungunya fever following the importation of initial cases. As of October 11, more than 20,000 cases have been reported in southern China, indicating an increasing risk of chikungunya fever [35]. It should be noted that significant differences exist between Ruili and cities in Guangdong Province in terms of ecological environment, including the predominant *Aede*s species (potential presence of *Ae. aegypti* in Ruili vs. *Ae. albopictus* in Guangdong cities) and climate conditions (tropical monsoon in Ruili compared to subtropical monsoon in Guangdong cities). Furthermore, Ruili is a border city where imported cases are primarily associated with cross-border workers, while most cities in Guangdong Province are inland, with imported cases largely stemming from international travelers. These disparities suggest that the epidemiological patterns may differ between the two regions and warrant further investigation.

Moreover, chikungunya fever, a self-limiting illness typically presenting with mild symptoms, is frequently overlooked by patients, leading to delayed medical consultations. The combination of delayed case reporting and high patient mobility complicates source management and impede effective epidemic control efforts. Thus, strengthening the competencies of public health professionals in chikungunya prevention and control is of critical importance. Additionally, comprehensive and targeted public awareness campaigns are essential to promote timely healthcare-seeking behavior and early case detection, thereby facilitating transmission interruption and improves overall disease control.

Some limitations in our study should be acknowledged. First, the comprehensive study was based outbreak data with limited case numbers and a small study area, which may affect the comprehensiveness of the factor analysis. For instance, the panel logistic regression model indicated a very large effect of the occurrence of local cases in the previous week on the current occurrence of local cases (OR = 19.728), suggesting potential temporal autocorrelation in the transmission dynamics across time periods. Second, reliance on mosquito surveillance data may introduces potential biases in vector density estimates due to incomplete spatial coverage of monitoring areas. Third, although viral RNA can be detected in serum samples during the acute phase of CHIKV infection, serological methods are more appropriate for diagnosis following the peak of viremia. In this outbreak investigation, all patients underwent RT-PCR testing, while only a small subset underwent serological testing, likely leading to an underestimation of the total number of CHIKV infections. Moreover, the ecological mechanisms underlying the lagged effects of meteorological variables on CHIKV transmission warrant further investigation.

## Conclusions

In conclusion, based on an in-depth analysis of the ecological and environmental factors associated with the 2019 chikungunya outbreak in Ruili City, this study identified the occurrence of imported cases, population density, and percentage coverage of grassland as key determinants influencing the occurrence and extent of the outbreak. Given that border cities are particularly vulnerable to disease importation targeted interventions should be implemented during peak transmission periods, focusing on strengthening surveillance at entry points and enhancing vector control measures. Priority should be given to densely populated areas where transmission risk is elevated. Meanwhile, sustained public health education campaigns are recommended to improve community awareness and promote adherence to preventive practices.

## Supplementary Information

Below is the link to the electronic supplementary material.

Supplementary Material 1

## Data Availability

The datasets used and/or analyzed during the current study are available from the corresponding author on reasonable request.
