# Supplementary material for: The ecological factors contributing to the chikungunya outbreak in Ruili, a border city in Yunnan Province, China
Source: BMC Infect Dis. 2025 Nov 24;25:1643. doi: 10.1186/s12879-025-12056-1 (PMC12642045; doi:10.1186/s12879-025-12056-1)
Supplement: Supplementary file 1 — Supplementary Material 1 [file 12879_2025_12056_MOESM1_ESM.docx]

**Supplementary appendix**

**Supplement to: The ecological factors contributing to the chikungunya outbreak in Ruili, a border city in Yunnan Province, China.**

**Additional tables:**

**Table S1: The Spearman’s rank correlation analysis of weekly Breteau index and meteorological factors in Ruili City from 2018 to 2019.**

| **Variable** | **Lag** | **r*** | ***P* value** |
| --- | --- | --- | --- |
| Temperature | 0 | 0.487 | <0.001 |
|  | 1 | 0.569 | <0.001 |
|  | 2 | 0.639 | <0.001 |
| Relative humidity | 0 | 0.749 | <0.001 |
|  | 1 | 0.716 | <0.001 |
|  | 2 | 0.656 | <0.001 |
| Precipitation | 0 | 0.590 | <0.001 |
|  | 1 | 0.631 | <0.001 |
|  | 2 | 0.640 | <0.001 |

*Spearman’s rank correlation.

**Table S2: The ecological efficiency of local chikungunya transmission at the township-level in Ruili: a Poisson regression analysis using data from selected townships with Breteau Index records.**

| **Variable (unit)** | **Univariate analysis** | | |  | **Multivariate analysis** | | |
| --- | --- | --- | --- | --- | --- | --- | --- |
|  | **Crude IRR** | **95% CI** | **P value** |  | **Adjusted IRR** | **95% CI** | **P value** |
| The occurrence of imported cases (categorical) | 4.800 | (2.492, 9.247) | <0.001 |  | 2.782 | (1.352, 5.722) | 0.005 |
| Population density (1000 per km^2^) | 1.793 | (1.503, 2.139) | <0.001 |  | 1.477 | (1.160, 1.881) | 0.002 |
| NDVI | 0.024 | (0.007, 0.087) | <0.001 |  |  |  |  |
| Elevation (m) | 0.994 | (0.992, 0.996) | <0.001 |  |  |  |  |
| ^#^BI | 0.942 | (0.907, 0.979) | 0.002 |  |  |  |  |
| Land cover (%) |  |  |  |  |  |  |  |
| Percentage coverage of grassland | 1.133 | (1.084, 1.185) | <0.001 |  | 1.120 | (1.063, 1.180) | <0.001 |
| Percentage coverage of built-up land | 1.016 | (1.009, 1.023) | <0.001 |  |  |  |  |
| Percentage coverage of shrub | 0.000 | (0.000, 0.146) | 0.02 |  |  |  |  |
| Percentage coverage of cropland | 1.000 | (0.993, 1.008) | 0.844 |  |  |  |  |
| Percentage coverage of forest | 0.983 | (0.975, 0.991) | <0.001 |  |  |  |  |
| Percentage coverage of water body | 1.295 | (1.166, 1.439) | <0.001 |  |  |  |  |

Abbreviations: CI, confidence interval; IRR, incidence rate ratio; NDVI, Normalized Difference Vegetation Index; BI, Breteau index.

^#^The average at the township level during the study period.

**Additional figures:**


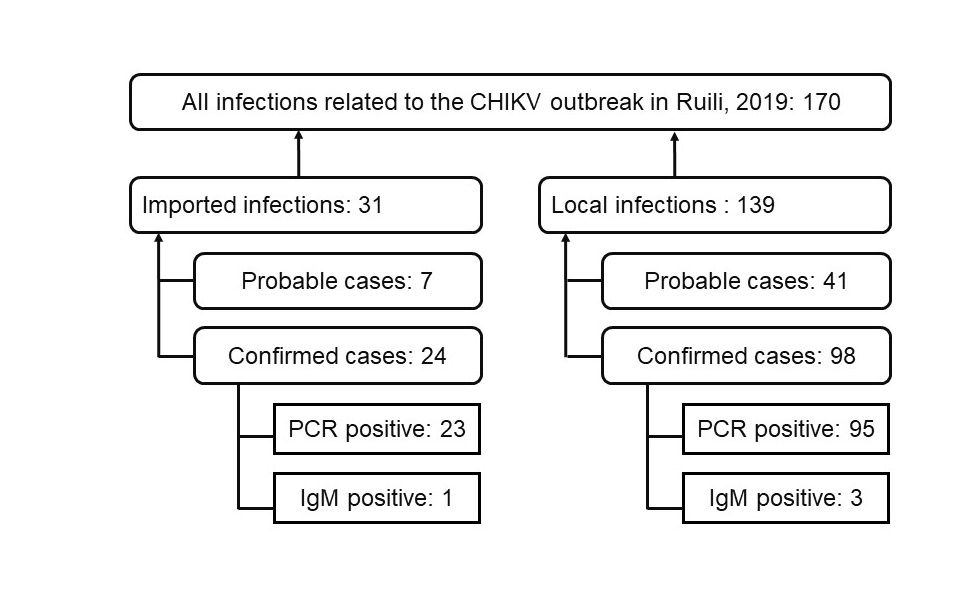


**Figure S1:** **CONSORT-style diagram for the all infections related to the CHIKV outbreak in Ruili, 2019.**


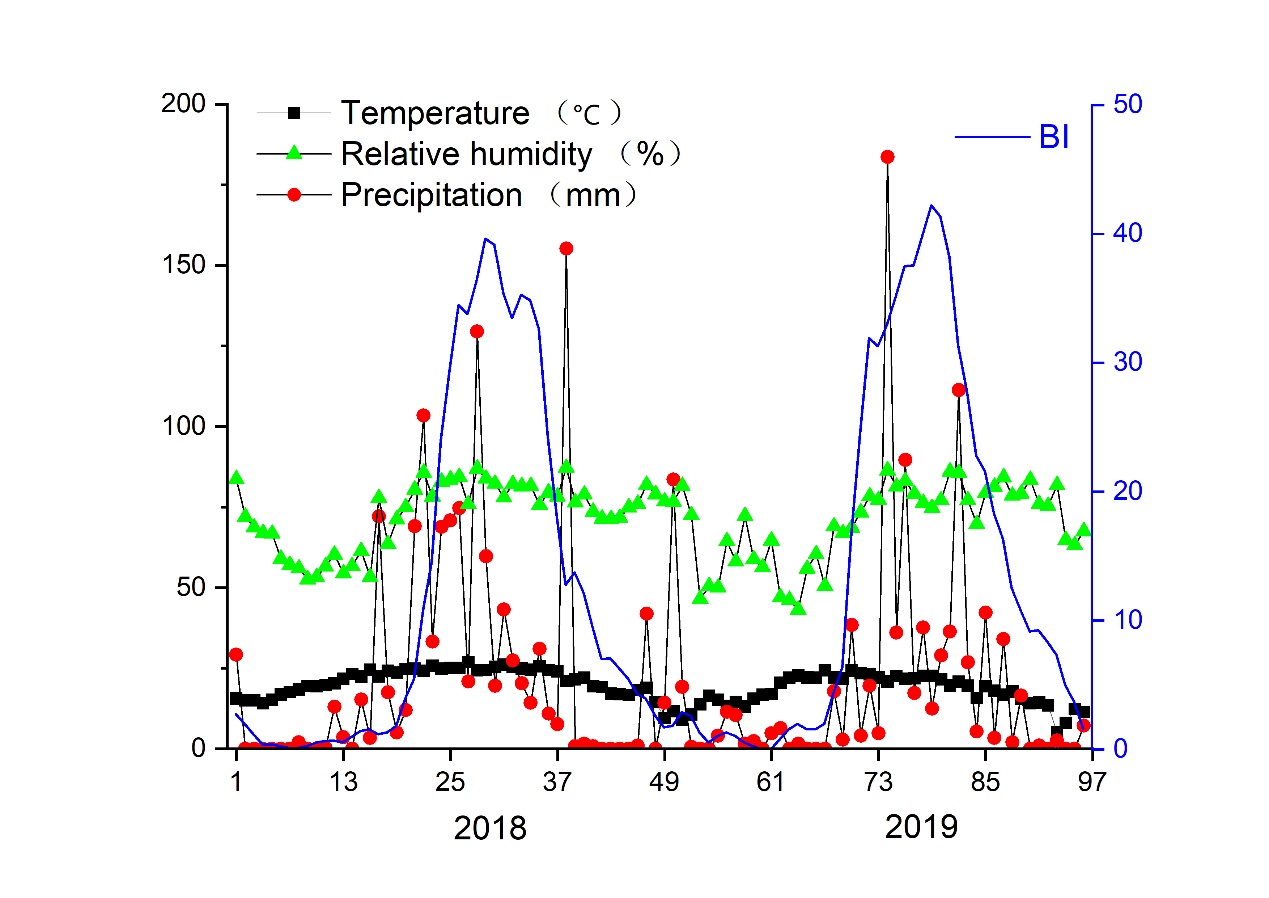


**Figure S2: The temporal dynamic of weekly Breteau index (BI) and meteorological factors in Ruili City from 2018 to 2019.**
